# Dispersion of the Vancomycin Resistance Genes *van*A and *van*C of *Enterococcus* Isolated from Nile Tilapia on Retail Sale: A Public Health Hazard

**DOI:** 10.3389/fmicb.2016.01354

**Published:** 2016-08-26

**Authors:** Kamelia M. Osman, Mohamed N. Ali, Ismail Radwan, Fatma ElHofy, Ahmed H. Abed, Ahmed Orabi, Nehal M. Fawzy

**Affiliations:** ^1^Department of Microbiology, Faculty of Veterinary Medicine, Cairo University, GizaEgypt; ^2^Department of Fish Diseases and Management, Animal Health Research Institute, GizaEgypt; ^3^Department of Bacteriology, Mycology and Immunology, Faculty of Veterinary Medicine, Beni-Suef University, Beni-SuefEgypt; ^4^Department of Microbiology, Faculty of Veterinary Medicine, Benha University, BenhaEgypt

**Keywords:** nile tilapia, antibiotic resistance phenotype, *esp*, *van*A and *van*C genes, *E. faecalis*, E. gallinarum

## Abstract

Although normally regarded harmless commensals, enterococci may cause a range of different infections in humans, including urinary tract infections, sepsis, and endocarditis. The acquisition of vancomycin resistance by enterococci (VRE) has seriously affected the treatment and infection control of these organisms. VRE are frequently resistant to all antibiotics that are effective treatment for vancomycin-susceptible enterococci, which leaves clinicians treating VRE infections with limited therapeutic options. With VRE emerging as a global threat to public health, we aimed to isolate, identify enterococci species from tilapia and their resistance to *van*-mediated glycopeptide (*van*A and *van*C) as well as the presence of enterococcal surface protein (*esp*) using conventional and molecular methods. The cultural, biochemical (Vitek 2 system) and polymerase chain reaction results revealed eight *Enterococcus* isolates from the 80 fish samples (10%) to be further identified as *E. faecalis* (6/8, 75%) and *E gallinarum* (2/8, 25%). Intraperitoneal injection of healthy Nile tilapia with the eight *Enterococcus* isolates caused significant morbidity (70%) within 3 days and 100% mortality at 6 days post-injection with general signs of septicemia. All of the eight *Enterococcus* isolates were found to be resistant to tetracycline. The 6/6 *E. faecalis* isolates were susceptible for penicillin, nitrofurantoin, gentamicin, and streptomycin. On the other hand 5/6 were susceptible for ampicillin, vancomycin, chloramphenicol, and ciprofloxacin. The two isolates of *E. gallinarum* were sensitive to rifampicin and ciprofloxacin and resistant to vancomycin, chloramphenicol, and erythromycin. Molecular characterization proved that they all presented the prototypic *van*C element. On the whole, one of the two vancomycin resistance gene was present in 3/8 of the enterococci isolates, while the *esp* virulence gene was present in 1/8 of the enterococci isolates. The results in this study emphasize the potential role that aquatic environments are correlated to proximity to anthropogenic activities in determining the antimicrobial resistance patterns of *Enterococcus* spp. recovered from fish in the river Nile in Giza, Elmounib, Egypt as a continuation of our larger study on the reservoirs of antibiotic resistance in the environment.

## Introduction

Some of the features that have made tilapia an advantageous fish species for culture are: rapid growth rate, firm and white muscle, the ability to survive in poor water conditions, and high reproductive success with limited requirements during incubation (Nandlal and Pickering, 2004). They belong to the base of the food chain, which makes them suitable to consume low cost and wide range of food sources with reduced ecological impact (Beveridge and Baird, 1998). In Egypt, the inland waters capture reached 250,196 in 2013 (FAO, 2015-2016). Egypt Aquaculture Production in 2010 was 900,000 tons of which Nile tilapia constituted more than 55% of the total production (FAO, 2015-2016) and in 2008, Egypt produced 13.8% of the world’s cultured tilapias (FAO, 2015-2016).

The global increase in intensive fish farming has been accompanied by bacterial infections that are usually treated with antibiotics added to fish foodstuffs as therapeutic agents, prophylactics and growth promoters (Muñoz-Atienza et al., 2013) resulting in the emergence and spread of antibiotic-resistant bacteria in the aquaculture environment and increasing the risk of antibiotic resistance bacterial transfer to humans and in the alterations of the bacterial microbiota of the aquatic environment. The main problem with the use of antibiotics in aquaculture is that many of the antibiotics that are used are from antibiotic classes that are also used in the treatment of human bacterial infections. The problem of increasing resistance is an ongoing and evolving issue and to be recognized at the global level by several international organizations with the so-called Tripartite comprising the WHO, FAO and the OIE leading the discussions on critical needs for human and animal health, and on the relation between the usage of antimicrobials in animal production and the consequences for human health which led to draft the third revision of the “WHO List of Critically Important Antimicrobials for Human Medicine” (WHO, 2012b).

Enterococci are widely distributed in nature (Klare et al., 2003), also known to be present in the intestine of healthy fish, and are potential probiotic candidates in aquaculture. Enterococci have been known to be resistant to most antibiotics used in clinical practice (Lukášoá and Šustáčková, 2003) and have been implicated in severe human infections as a consequence of associated determinants of virulence and antimicrobial resistance with the vancomycin-resistant prevalence to the harbored *van*A gene (Palazzo et al., 2011). Clinically relevant enterococci (*Enterococcus faecalis*, and *E. gallinarum*, the *van*C-type resistance group) are frequent causes of nosocomial infections, like urinary tract infections and sepsis (Menichetti, 2005). Antibiotic resistance genes exist not only in nature and in humans but also in fish as an enormous reservoir of genes that can be transferred to human pathogens either by direct contact or indirectly through the consumption of contaminated fish. Examples of antibiotic resistance genes detected in aquaculture over the last 7 years are summarized in Rolain (Rolain, 2013). Since development of the second revision in 2009 (WHO, 2012a), the committee faced several new issues of which vancomycin-resistant enterococci was one of them. In 1993 the first non-human reservoir of *van*A vancomycin resistant *E. faecium* were identified (Pruden and Shore, 2011).

As vancomycin resistant by enterococci (VRE) are emerging as a global threat to public health, therefore, the objective of the study was to improve the understanding of antibiotic resistance through characterization of antibiotic-resistant VRE isolates associated with the fish retail market. Assessment and identification of the *Enterococcus* species was implanted by conventional and molecular (16S rRNA and ddl*_E.faecalis_*) assays isolated from tilapia and to evaluate the virulence impression *esp* which encodes for the *Enterococcus* surface protein (ESP), which participates in biofilm formation and is associated with colonization and persistence in the urinary tract promotes adhesion, colonization and evasion of the immune system, and to play some role in antibiotic resistance (Foulquie Moreno et al., 2006). The two vancomycin antimicrobial resistance genes *van*A (associated with a high level of inducible resistance to vancomycin and cross resistance to teicoplanin) and *van*C (mediated by the chromosomal *VANC1* gene, which is constitutively present in *E. gallinarum* conferring relatively low resistance levels to vancomycin and is not transferable) were also molecularly assayed.

## Materials and Methods

### Fish Collection and Sampling

Eighty random fish were sampled from five different market types: supermarkets, fish mongers and fish vendors, landing sites, wholesale markets. Fish were placed in a cooler containing ice and transported back to lab. The liver, kidney, brain, spleen and ascitic fluid were removed by dissecting the fish. Twenty-five grams of each sample was homogenized in 225 mL of bile esculin azide broth (BBL) in Stomacher Lab Blender 400 for 30 s from which 0.1 and 0.01 mL were spread onto the surface of bile esculin azide agar plates (BBL) and incubated at 45°C for 24 and 48 h. The initial dilutions were treated similarly. In order to guarantee the absence of contamination, typical enterococci colonies (black) on bile esculine azide agar were streaked onto blood agar (Oxoid) plates prepared with 5% of defibrinated sheep blood and incubated at 37°C for 24 and 48 h. The phenotypic characterization of bacterial isolates was studied to determine their colony morphology, cell morphology, motility, Gram stain and to be finally characterized using the Vitek 2 system (bioMerieux SA, Marcy I’Etoile, France).

### Molecular Identification of *Enterococcus* Species

Identification of presumptive enterococci was confirmed using the polymerase chain reaction (PCR): one mL of culture was aseptically spreaded on MRS agar plates and was incubated at 25°C for 3–5 days. Eventually, the forming colonies were transferred onto another new MRS agar plates until discrete colonies were obtained. Each discrete colony was further subcultured for several times to ensure that a pure culture was obtained. The virulent strain was identified by biochemical characterization based on the ability of utilization of different carbon sources by Vitek 2 (bioMerieux, Lyon, France). The genomic DNA was extracted using Wizard Genomic DNA Purification Kit (Promega, USA) and used as a template for PCR using *Enterococcus* genus-specific primers to amplify the conserved sequence of the 16S rRNA gene (Karsidani et al. (2010) that yield a 112 bp PCR product as outlined in **Table 1**. PCR products were analyzed by electrophoresis on 1–2% (w/v) agarose (Pronadisa, Madrid, Spain) gels stained with Gel red (Biotium, Fremont, CA, USA), and visualized with the Gel Doc 1000 documentation system (Bio-Rad, Madrid, Spain).

**Table 1:** Oligonucleotide primers sequences and size of the PCR-targeted products.

Microorganism	Target gene	bp fragment	Primer sequence (5′–3′)	Annealing temp (°C)	Reference
*E. faecalis/E. gallinarum*	16S rRNA (Genus -specific primers)	112 bp	F TAC TGA CAA ACC ATT CAT GAT G	59 (*E. faecalis*)	Karsidani et al., 2010
			R AAC TTC GTC ACC AAC GCG AAC	50 (*E. gallinarum*)	
	*vanA* (Glycopeptide resistance genotype Vancomycin resistance)	732 bp	F GGG AAA ACG ACA ATT GC	59 (*E. faecalis*)	Biavasco et al., 2007; Karsidani et al., 2010
			R GTA CAA TGCG GCC GTTA	50 (*E. gallinarum*)	
	*vanC1* (Glycopeptide resistance genotype Vancomycin resistance)	822 bp	F GGT ATC AAG GAA ACC TC	59 (*E. faecalis*)	
			R CTT CCG CCA TCA TAG CT	50 (*E. gallinarum*)	
*E. faecalis*	*ddl_E.__faecalis_* (Species-specific primers)	941 bp	F ATC AAG TAC AGT TAG TCT	55	
			R ACG ATT CAA AGC TAA CTG		
	*esp* (virulence factor)	932 bp	F TTG CTA ATG CTA GTC CAC GACC	55	
			R GCG TCA ACA CTT GCA TTG CCG AA		

### Detection of *In vivo* Potential Virulence

#### Experimental Infection

The study was approved by the Cairo University ethical committee for animal research, Egyptian animal welfare agency, Cairo, Egypt. The procedures used for animal care and housing were in accordance with the U.S. Department of Agriculture through the Animal Welfare Act (7USC 2131) 1985 and Animal Welfare Standards incorporated in 9 CFR Part 3, 1991.

In order to determine the virulent capacities of the eight isolates, infectivity trials were conducted through the intraperitoneal injection of each of the eight *Enterococcus* isolates using Nile tilapia as a model. Prior to challenge, the fish were randomly sampled to be subjected to microbiological analysis which indicated that they were free of enterococci. As a control, *E. faecalis* strains V583 and OG1RF, as well as *E. faecium* 64/3 were used. The procedure carried out by Martins et al. (2008) was adopted. Eighty fish weighing 55.0 ± 5.0 g were distributed in 12 aerated aquaria of 100 cm × 40 cm × 25 cm and acclimatized for 10 days before assay. During the course of the experiments (21 days) a daily 50% water renovation was provided and feeding the fish with commercial diet. During this period, the water temperature was maintained at 25.0 ± 0.2°C, pH 7.0 ± 0.2 and ammonia 0.5 ± 0.1 mg L^-1^. The colonies confirmed as *Enterococcus* sp. by PCR (*E. faecalis, E. gallinarum*) were prepared as an inoculum of the bacteria diluted in tubes containing infusion of heart and brain (BHI; Difco, Detroit, MI, USA) to reach the concentration of 1 × 10^7^ CFU/mL estimated by serial-dilution method (1:10). The experiment was done by challenging the fish with 10^7^ CFU/ml of each of the live *Enterococcus* species (*E. faecalis, E. gallinarum*). The 80 fish were divided into eight groups, each group comprised of 10 fish was intraperitoneally challenged with each individual *Enterococcus* isolate. The control fish (unchallenged group) received 0.1 μl of saline solution (0.85% NaCl) through i.p. injection. The clinical signs of the fish were observed continuously within 24 h duration of the experiment and fish mortality was daily checked during this period. The liver, brain and kidney from the dead fish were collected and microbiologically analyzed for re-isolation and identification of the inoculated strain by bacterial culture and PCR. The degree of virulence was expressed as lethal dose 50% (LD50) calculated as described by Reed and Müench (1938).

#### Bacteria Culture

The swab from the organs that were collected four hourly were immediately streaked onto the blood agar plates and incubated at 30°C for 24 h. Gram staining were performed to identify Gram-positive cocci in chain or paired and catalase test negative organisms. Finally, the colonies were further characterized using the Vitek 2 system (bioMerieux).

#### Polymerase Chain Reaction

For confirmation of *E. faecalis* and *E. gallinarum*, total cellular DNA was extracted using Wizard Genomic DNA Purification Kit (Promega, USA) according to manufacturer’s protocol. The extracted DNA was then further evaluated by PCR for *E. faecalis* and *E. gallinarum* -specific section of 16S rRNA. PCR products were analyzed by electrophoresis as previously indicated.

### PCR Detection of the Potential Virulence Factor *esp* in *E. faecalis* and *E. gallinarum*

Detection of the *esp* (enterococcal surface protein) gene in the eight enterococci was performed by PCR (**Table 1**). The positive control strain for detection of potential virulence factor was *E. faecalis* P36 for *esp*. PCR-amplifications was performed from total bacterial DNA obtained using the Wizard DNA Purification Kit (Promega, Madrid, Spain) in 25 μl reaction mixtures with 1 μl of purified DNA, 0.7 μM of each primer, 0.2 mM of each dNTP, buffer 1×, 1.5 mM MgCl_2_ and 0.75 U of Platinum Taq DNA polymerase (Invitrogen, Madrid, Spain). PCR products were analyzed by electrophoresis as previously indicated.

#### Determination of Antibiotic Resistance Phenotype

Antibiotic susceptibility of the eight enterococci was determined by overlaying antibiotic-containing disks (Oxoid) on Diagnostic Sensitivity Test Agar (Oxoid) previously seeded with approximately 1 × 10^5^ CFU/ml of each enterococcal isolate. The antibiotics tested and their concentrations are listed in **Table 2**. Inhibition zone diameters were measured after overnight incubation of the plates at 37°C. Resistance phenotypes were recorded as recommended by the CLSI (2011). All isolates were subjected to the minimal inhibitory concentration (MIC) to vancomycin and the assessments were conducted using the microbroth dilution method as previously recommended (CLSI, 2011). *E. faecalis* ATCC 29212 and *Staphylococcus aureus* ATCC29213 were used as quality control strains.

**Table 2:** Antibiotic interpretation for *Enterococcus* species.

On the WHO’s critically important antimicrobials list (WHO, 2012b)	Antibiotic disk	Concentration	Antibiotic interpretation							
			*E. faecalis* (*n* = 6)				*E. gallinarum* (*n* = 2)			
			Sensitive		Resistant		Sensitive		Resistant	
			*n*	%	*n*	%	*n*	%	*n*	%
**Penicillins**										
Critically important	Penicillin	10 μg	6	100	0	0	1	50	1	50
Critically important	Ampicillin	10 μg	5	83.3	1	16.7	1	50	1	50
**Glycopeptides**										
Critically important	Vancomycin	30 μg	5	83.3	1	16.7	0	0	2	100
**Macrolides**										
Critically important	Erythromycin	15 μg	2	33.3	4	66.7	0	0	2	100
**Tetracyclines**										
Highly important	Tetracycline	30 μg	0	0	6	100	0	0	2	100
**Fluoroquinolones**										
Critically important	Ciprofloxacin	5 μg	5	83.3	1	16.7	2	100	0	0
**Nitrofurantoins**										
Important	Nitrofurantoin	300 μg	6	100	0	0	1	50	1	50
**Phenicols**										
Highly important	Chloramphenicol	30 μg	5	83.3	1	16.7	0	0	2	100
**Ansamycins**										
Critically important	Rifampicin	5 μg	2	33.3	4	66.7	2	100	0	0
**Aminoglycosides**										
Critically important	Gentamicin	10 μg	6	100	0	0	1	50	1	50
Critically important	Streptomycin	10 μg	6	100	0	0	1	50	1	50

#### Detection of Genetic Determinants of Antimicrobial Resistance

Total bacterial DNA was extracted by the GenElute^TM^ Bacterial Genomic DNA Kit (Sigma-Aldrich, St. Louis, MO, USA). Vancomycin-resistant isolates were studied for the presence of the glycopeptides resistance genes *Van*A and *Van*C1 by PCR (**Table 1**).

## Results

**FIGURE 1:**
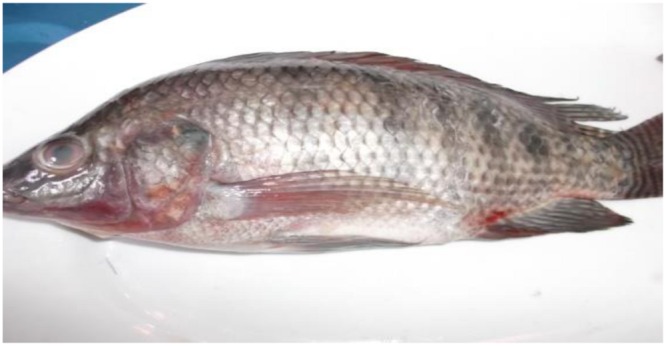
**Tilapia fish showing externally eye opacity, dark skin discoloration in the form of strips, congested base at the pelvic fin, hemorrhagic pelvic fin, and distended abdomen**.

The clinical picture and postmortem lesions of the experimentally infected tilapia were seen as: external lesions (**Figure 1**): eye lesions: in the form of unilateral or bilateral eye redness/opacity; Skin lesions: detached scales, extensive skin congestion, ulcers, hemorrhage, or dark discoloration in the form of strips; Fins: congestion at the base of the fins, or even hemorrhagic; Abdomen: slightly distended in some cases; Anal opening: congested with protruded anal opening.

**FIGURE 2:**
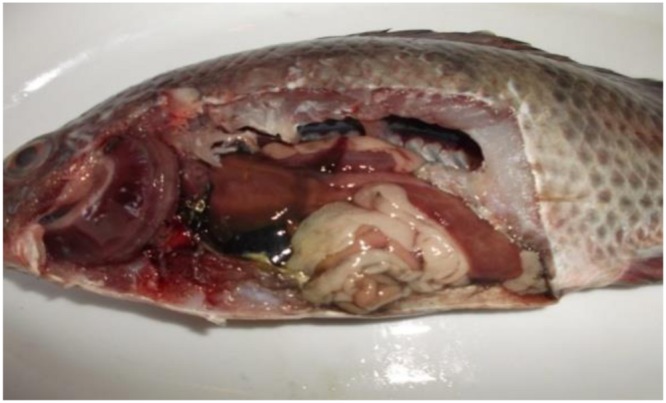
**Tilapia fish postmortem internal findings revealed typical signs of septicemia in which severely congested gill, kidney, spleen, intestine and heart, dark gall bladder, in addition to hemorrhagic ascites, friable mottled liver, muscle redness, and parts of the intestine were devoid of food**.

Postmortem lesions (**Figure 2**): general signs of septicemia; Muscle: redness; Kidney: renal congestion; Liver: friable, pale and congested, dark gall bladder; Spleen: enlarged and congested; Heart: congested; Intestine: intestinal congestion and devoid of food; Gill: congested; Abdominal cavity: hemorrhagic ascites.

Biochemical identification of the *Enterococcus* species was successfully implemented through the Vitek 2 system. The cultural, biochemical (Vitek 2 system) and PCR results revealed eight *Enterococcus* strains from the 80 fish samples (10%) to be further identified as *E. faecalis* (6/8, 75%) and *E gallinarum* (2/8, 25%). Although the eight isolates were non-hemolytic, they were then proven to be pathogenic through the *in vivo* experimental infection. Intraperitoneal injection of healthy Nile tilapia with the eight *Enterococcus* isolates caused significant morbidity (70%) within 3 days and 100% mortality at 6 days post-injection with general signs of septicemia.

The antibiotic sensitivity test for the *Enterococcus* species showed that 6/6 of the *E. faecalis* were susceptible for penicillin, nitrofurantoin, gentamicin, and streptomycin, while all isolates (8/8) were resistant to tetracycline (**Table 2**). Only 1/6 *E. faecalis* isolates was resistant to vancomycin. On the other hand, 5/6 of the *E. faecalis* were susceptible for ampicillin, vancomycin, chloramphenicol, and ciprofloxacin. The lowest rate of sensitivity was observed for rifampicin and erythromycin (2/6). The 2/2 isolates of *E. gallinarum* were sensitive to rifampicin and ciprofloxacin, but resistant to vancomycin, chloramphenicol, and erythromycin. Among the eight isolates, 1/2 *E. gallinarum* and 4/6 *E. faecalis* were resistant to at least three antibiotics. High level of multidrug resistance to critically important antibiotics was detected in *E. gallinarum* strains (6/11 of *E. gallinarum* versus 5/11 of *E. faecalis*). Vancomycin resistant isolates exhibited high and consistent MIC values (≤230 μg/ml).

As indicated in **Table 3**, the *van*C and *van*A resistance genes were present in combination in one of the two *E. gallinarum* isolates (50%), while the second isolate carried the *van*C gene only (50%). The *E. faecalis* (six isolates), exhibited the following pattern: the *van*A gene was found in 1/6 isolates, while none of the *van*A and *van*C were indicated in 5/6 isolates.

**Table 3:** Genetic profile of isolated bacteria.

Bacterial isolates	Total *n* = isolates	Genetic profile	*n* = of +ive isolates	%
*E. gallinarum*	2	16S rRNA+ *Van*C	1	50
		16S rRNA+ *Van*A+ *Van*C	2	100
*E. faecalis*	6	16S rRNA + *Van*C+ ddl*_E.faecalis_*	0	0
		16S rRNA + ddl*_E.faecalis_* + *esp*	1	16.6
		16S rRNA + ddl*_E.faecalis_* + *Van*A	1	16.6
		16S rRNA + ddl*_E.faecalis_*	4	50

*Detection of *in vivo* potential virulence: experimental infection.*

The virulence gene *esp* was found in 1/6 of the *E. faecalis* isolates and absent from the 2/2 *E. gallinarum* isolates.

On the whole, one of the two vancomycin resistance gene was present in 37.5% of the enterococci isolates (3/8), while the *esp* virulence gene was present in one out of the eight enterococci isolates (12.5%).

## Discussion

Public health impacts from antibiotic use in agriculture and aquaculture have already drawn much attention in the last decade (Heuer et al., 2009; Marshall and Levy, 2011; Kuehn, 2012; WHO, 2012a). Therefore, it is important to monitor the occurrence of antibiotic resistant bacteria in surface waters used for drinking and irrigation purposes since these bacteria are able to spread through food to humans.

For species differentiation, the motility and pigment tests were performed to phenotypically distinguish among species where *E. faecalis* was non-motile whereas *E. gallinarum* was motile (CDC, 2010). *In vivo* experimentation, using local strains of Nile tilapia, was utilized to elucidate and assess the *in vivo* survival and pathogenic potential of the eight enterococci (*E. faecalis*, *n* = 6 and *E. gallinarum, n* = 2) strains. Growth *in vitro* did not differ between these two strains. In the Nile tilapia model, the eight isolates were rapidly lethal and pathogenic. Koch’s postulates confirmed *E. faecalis* and *E. gallinarum* as the causative agents of enterococcosis among the captured Nile tilapia in Egypt.

Various lists of critically important antibiotics, such as those published by the WHO (2012b) are also given for prophylaxis in industrial fish aquaculture (Cabello, 2006) to fight disease or/and growth promoters to end up on our table as seafood. This has resulted in an increase of both antibiotic resistance bacteria in fish pathogens and in antibiotic resistance in the environment (Cabello, 2006) which must consequently in an act to prioritize the importance of strict regulatory use of antibiotics. Researchers at the Arizona State University’s Biodesign institute examined antibiotic use in shrimp, salmon, catfish, trout, tilapia and swai, originating from 11 countries (Bangladesh, Canada, Chile, China, Indonesia, Mexico, Panama, Scotland, Thailand, USA, and Vietnam). Of the 47 antibiotics evaluated, they discovered traces of just five (Done and Halden, 2015) of which, oxytetracycline was the most prevalent in the study samples. They hypothesized that the very low number of antibiotics (5/47) recovered was because antibiotics have a period of time between when they are administered and when the fish is processed (Done and Halden, 2015). However, regulations in this area vary between countries (Luening, 2015). As in Vietnam (Pham et al., 2015), the Egyptian Farmers’ decision-making processes about antimicrobial use, are influenced by biased sources of information, such as drug manufacturers, sellers and by financial incentives.

Muñoz-Atienza et al. (2013) found that, the percentage of enterococcal strains showing acquired antibiotic resistance was 68%. Interestingly, our results found *E. faecalis* with a high percentage of resistance to ciprofloxacin and/or norfloxacin, rifampicin, and glycopeptides with a very low resistance (5%) to erythromycin and tetracycline. In spite of the high prevalence of acquired antibiotic resistance found in their enterococci of aquatic origin, they showed low incidence or absence of resistance to the clinically relevant antibiotics vancomycin, ampicillin, penicillin and gentamicin, which is in agreement with previous studies but in contrast to our findings. Fortunately, our observations indicated a low level of antibiotic resistance in the *E. faecalis* isolates making it a non-feared infectious agent in intensive care ward (Higuita and Huycke, 2014).

From the five recognized genes, *van*A, *van*B, *van*C, *van*D, and *van*E contributing to vancomycin resistance in enterococci. (CDC, 2010), we focused on two genes *van*A and *van*C. These genes were previously studied in Egypt in large animals and poultry so it was our opinion to extend them to fish. In addition to motility and pigment tests, an organism’s susceptibility profile also helps differentiate *van*A isolates from *van*C isolates (CDC, 2010). Identification of VRE to species level aids in confirming whether an isolate has intrinsic (*van*C) or acquired resistance (*van*A). The *van*A is mediated by newly acquired gene clusters not previously found in enterococci and described primarily in *E. faecalis* and *van*A-resistant strains possess inducible, high-level resistance to vancomycin. On the other hand, the *van*C resistance phenotype was described in *E. gallinarum*, which demonstrate intrinsic, low-level resistance to vancomycin. Knowledge of the type of resistance is critical for infection control purposes as *van*A is transferable and can spread from organism to organism while in contrast, *van*C genes is not transferable and has been associated less commonly with serious infections or associated with outbreaks (CDC, 2010).

The *van*A resistance gene seems to be widespread in enterococci of natural ecosystems, in addition to clinical settings, and it would be interesting to track the routes of dissemination of these resistant microorganisms that have great importance in public health (Barros et al., 2012). Vancomycin resistance in enterococci isolated from food has a variable global pattern and the vancomycin resistant strains appears to be disparate according to the geographic region where they emerge (Franz et al., 2001; Giraffa, 2002; Gomes et al., 2008; Van Tyne and Gilmore, 2014). In Europe, this divergence is attributed to the use of antimicrobial agents as growth promoter whereas in the United States, it is attributed to the wide hospital usage of vancomycin (Van Tyne and Gilmore, 2014).

Enterococci should be considered a significant zoonotic pathogen and a possible reservoir of genes encoding resistance potentially transferred to other bacterial species (Kuhn et al., 2000) as there is ample evidence that many antibiotic resistance determinants found in pathogenic human bacteria have a fish origin (Cabello, 2006; Penders et al., 2013; Rolain, 2013). Spreading of resistance genes is a high risk as they are highly persistent and do not disappear from aquaculture sites, even after several years after eliminating the use of antibiotics (Tamminen et al., 2011). Thus, resistant bacteria from the fish that are consumed by humans, as well as direct contact between humans and fish, can be a source of antibiotic resistance gene transfer (Schjorring and Krogfelt, 2011). It must be emphasized that, 5/6 of the *E. faecalis* isolates were recovered from the river Nile in Giza ElMounib while the remaining one isolate was from an aquaculture site located in ElTal Elkebir in the Delta. This opens the discussion as to the significance and seriousness of the ElMounib location. In Egypt, poverty levels, overcrowdness, informal settlements in such areas as in ElMounib are issues that complicate the problem of antibiotic resistance where the presence of pathogenic enterococci in sewage-contaminated river water where bacterial contamination of surface water and particularly contamination with fecal-derived bacteria, has long been a water quality issue owing to the potential for disease transmission (Kinge et al., 2010; Romanis, 2013). It has been reported in the literature that surface waters have become a major reservoir of multi-antibiotic resistant pathogenic bacteria due to contamination by agricultural waste, animal excreta, the effluent water used for irrigation purposes and sewage disposal (Lupo et al., 2012). Consequently, the enterococci carrying ARG disseminated in the River Nile via waste water or run-off from livestock facilities and agriculture in the area under investigation contaminates the Nile tilapia.

The elevated levels of ARGs in aquatic environments are correlated to proximity to anthropogenic activities with special reference to environmental impact of agricultural effluent, irrigation water and fishing (Berglund, 2015), large-scale industrial agricultural facilities, which raise food animals at high-density and using antibiotics for treatment or as growth promoters (Burkholder et al., 2007; Gilchrist et al., 2007; Greger and Koneswaran, 2010). Similarly, impacts from large scale and widespread antibiotic use in aquaculture need to be re-assessed (Knapp et al., 2010; Weir et al., 2012). In aquaculture, fish infections are treated through the administration of antibiotics directly into the water, avoiding any kind of purification processes (Kümmerer, 2009). The presence of these organisms in the fish samples is disquieting as this could lead to major human health problems. This emphasizes the need for constant evaluation of the wastewater treatment effluents to ensure compliance to set guidelines (Olaniran et al., 2015).

The fact that the *E. faecalis* and *E. gallinarum* strains eval- uated in this work that were lacking the *esp* gene, might be related with their non-clinical origin and absence of ampicillin resistance. This was observed in previous studies which reported that *esp* are more common in ampicillin-resistant/vancomycin-resistant *E. faecium* (VREF) than in ampicillin-susceptible/VREF strains (Vankerckhoven et al., 2004; Muñoz-Atienza et al., 2013). In this context, the increase in the incidence of VREF at hospital settings has been attributed mainly to the spread of ampicillin-resistant VREF exhibiting *esp* (Klare et al., 2005; Werner et al., 2008; Muñoz-Atienza et al., 2013). The discrepancies found when comparing our data with those obtained in other studies could be due to several factors: (i) differences in the antibiotics used and sources isolated, (ii) to the resistance mechanism of *Enterococcus* spp. under antibiotic selection pressure induced by antibiotic use differing with the aquaculture environment, (iii) It could be that various global factors are relevant to the antibiotic resistance of bacteria, (iv) the differences among fish groups might be due to various non-antibiotic factors (e.g., cadmium) driving the co-selection of antibiotic resistance according to different methods of fish management.

Our PCR results showed that *E. faecalis* and *E. gallinarum* strains evaluated in this work showed that, the *esp* which encode a cell wall-associated protein involved in immune evasion, was absent in the two *E. gallinarum* strains and found in 1/6 of the *E. faecalis* strains isolated. The same gene was not found in any of the tested Lactic Acid Bacteria by Muñoz-Atienza et al. (2013). As screening for the presence of virulence genes to evaluate the potential virulence of enterococci could be speculative, empirical testing with a disease challenge was conducted. Experimental pathogenicity test *in vivo* proved that, the eight isolates of the *Enterococcus* species were pathogenic. The infection or pathogenicity process of *Enterococcus* spp. is very complex and is said to involve different virulent and pathogenicity factors which either act together or separately at different stages of infection (Al-Bahry et al., 2014). Several authors have analyzed a number of virulence factors in *E. faecalis*, including a structurally novel toxin, a surface protein, *esp* (Shankar et al., 1999), which contributes to colonization of the bladder in a model of urinary tract infection (Shankar et al., 2001) and confers to the biofilm production capability of enterococci (Toledo-Arana et al., 2001).

## Conclusion

The present study supports the view that there is a risk of transfer of resistant bacteria to humans from consumption of fish and that follow-up studies are required to: (a) investigate the extent of antibiotic use in Egyptian aquaculture farms, (b) to determine the molecular basis of antimicrobial resistance to the different antibiotics, (c) the potential for transfer of resistance genes from aquaculture isolates to human pathogens, (d) some assessment of the risk of transfer of resistant organisms (or genes) to humans via food chain, and (e) the threats imposed by environmental contamination with antibiotic resistant bacteria. In addition to antibiotic resistance dissemination into the surrounding environment, the use of antibiotics in aquaculture facilities without proper sanitation techniques, the antibiotics can accumulate in the sediment at the bottom of aquaculture ponds for extended periods of time, creating constant pressure to bacterial populations that increase the risk of antibiotic resistance in bacterial pathogens found in the fish water environment (Baquero et al., 2008; Pruden and Shore, 2011) and to the personnel working in the facility. Therefore, subsequent action should be taken to enforce the restricted use of certain antibiotics that are critically important for the treatment of human illnesses.

## Author Contributions

KO developed the concept, designed experiments, collected, and analyzed data and prepared the manuscript; MA, IR, FH, and AA gave technical support and conceptual advice; AO and NF performed PCR assays. All authors discussed the results and implications and commented on the manuscript at all stages.

## Conflict of Interest Statement

The authors declare that the research was conducted in the absence of any commercial or financial relationships that could be construed as a potential conflict of interest.
